# Effect of Hemin on Brain Alterations and Neuroglobin Expression in Water Immersion Restraint Stressed Rats

**DOI:** 10.1155/2016/7825396

**Published:** 2016-03-17

**Authors:** Merhan Ragy, Fatma Ali, Maggie M. Ramzy

**Affiliations:** ^1^Physiology Department, Faculty of Medicine, Minia University, Misr-Aswan Road, El-Minia 61519, Egypt; ^2^Biochemistry Department, Faculty of Medicine, Minia University, Misr-Aswan Road, El-Minia 61519, Egypt

## Abstract

In the brain, the heme oxygenase (HO) system has been reported to be very active and its modulation seems to play a crucial role in the pathophysiology of neurodegenerative disorders. Hemin as HO-1 inducer has been shown to attenuate neuronal injury so the goal of this study was to assess the effect of hemin therapy on the acute stress and how it would modulate neurological outcome. Thirty male albino rats were divided into three groups: control group and stressed group with six-hour water immersion restraint stress (WIRS) and stressed group, treated with hemin, in which each rat received a single intraperitoneal injection of hemin at a dose level of 50 mg/kg body weight at 12 hours before exposure to WIRS. Stress hormones, oxidative stress markers, malondialdehyde (MDA), and total antioxidant capacity (TAC) were measured and expressions of neuroglobin and S100B mRNA in brain tissue were assayed. Our results revealed that hemin significantly affects brain alterations induced by acute stress and this may be through increased expression of neuroglobin and through antioxidant effect. Hemin decreased blood-brain barrier damage as it significantly decreased the expression of S100B. These results suggest that hemin may be an effective therapy for being neuroprotective against acute stress.

## 1. Introduction

Stress is any condition that impairs the balance of the organism physiologically or psychologically and the response to stress involves several neuro-hormonal consequences [1]. Stress-mediated effects on brain function depends on the type of stressor, and the development stage of the organism so different physical and psychosocial stresses are associated with various diseases such as mental disorders, anxiety and depression, as well as neurodegenerative diseases [2].

In response to stress, different molecular pathways become activated, including reactive oxygen species (ROS) production and oxidative stress, inflammatory pathways, and apoptotic cell death pathways. These pathways are also considered as major components of the pathophysiology of neurodegenerative disorders [3].

Heme oxygenase (HO) degrades heme to produce carbon monoxide (CO), iron (Fe^2+^), and biliverdin, which is rapidly converted to bilirubin. Three isoforms of HO were identified in the brain: inducible form (HO-1) and constitutive forms (HO-2 and HO-3). HO-1 and HO-2 may have different mechanisms to protect neurons from oxidative stress [4].

Previous studies have reported controversial effects of HO-1 induction, either deleterious or beneficial depending on the different neuroinflammatory models and various drug exposure methods [5–7]. HO-1 may confer neuroprotective or neurotoxic effects because of the balance between beneficial and toxic effects of heme and heme products [4].

Neuroglobin (Ngb) is the third generation of oxygen-carrying globin and expressed in the neurons system [8]. It has been proposed that Ngb could have a function similar to that of myoglobin and could serve to transport oxygen to neuronal mitochondria [9] so studies have suggested that Ngb is closely related to ischemic-hypoxic brain damage [10]. Upregulation of Ngb expression has been observed in response to various pathological conditions, including cerebral ischemia, hypoxia, and toxicity, and it has been revealed that hemin specifically induces upregulation of Ngb in nerve cells [11].

S100B protein, a calcium binding protein secreted by astrocytes, is involved in glial-to-neuron signaling as a part of response of glial cell to stress [12]. S100B does not penetrate the blood-brain barrier and therefore it is not found in peripheral blood under normal circumstances [13]. Head trauma with structural lesions causes a disruption of this barrier, which, in turn, produces measurable amounts of S100B in peripheral blood [14]. Therefore, measuring S100B mRNA expression in brain is proffered to be done in the present study.

With respect to an existence of these discrepancies, this study was directed to investigate the effect of WIRS with or without a HO inducer on the brain of male albino rats and if there is a relation between hemin, Ngb, S100B, catecholamines, corticosterone, and brain oxidative state in WIRS rats.

## 2. Materials and Methods

### 2.1. Animals

Adult male albino rats aged between 8 and 10 weeks were used throughout the present study. The rats were maintained under normal standard lighting and feeding (rat chow and water ad libitum) conditions. All the procedures followed with the rats were in accordance with our institutional guidelines. Animals were left to acclimatize to the environment for two weeks prior to inclusion in the experiment. The rats were randomly classified into the following three groups (10 rats each):

1. control group, in which each rat received 0.5 mL of hemin vehicle intraperitoneally 12 hours before the stress period and left undisturbed during the stress period,
2. water immersion restraint stress (WIRS) group, in which each rat received 0.5 mL of the hemin vehicle intraperitoneally 12 hours before being exposed to WIRS,
3. stressed hemin-treated group (WIRS + hemin), where each rat received hemin at a dose level of 50 mg/kg body weight intraperitoneally 12 hours before being exposed to WIRS [10].

### 2.2. Drug Protocol

Hemin (Sigma, UT, USA) was freshly dissolved in 0.1 mol/L NaOH adjusted pH 7.4 with 0.1 mol/L HCl and diluted with saline to required volume (vehicle). Hemin was prepared in darkness and protected from light [15]. Hemin dose was selected on the basis of previous studies [10, 16].

### 2.3. Water Immersion Restraint Stress (WIRS)

Each rat was immobilized on a wooden board by taping the four limbs with surgical tapes to specially prepared metal mounts and immersed up to the depth of the xiphoid process in water bath at 23°C for 6 hours [17] and then immediately decapitated after stress was relieved.

### 2.4. Sample Collection

All rats were sacrificed by decapitation immediately after experimental period. Blood samples were collected and centrifuged for 10 min at 5000 rpm. The obtained clear sera were stored at −80°C until required for determination of the following:

1. Corticosterone level was determined using spectrophotoflurometric method; the procedure essentially entails an extraction of free 11-hydroxycorticosteroids from serum, mainly cortisol and corticosterone, by methylene chloride followed by their condensation with an acidic fluorescence reagent. The induced fluorescence is measured at 510 nm after excitation at 450 nm [18].
2. Serum catecholamines (epinephrine and norepinephrine) were determined using spectrophotoflurometric method. Oxidation of catecholamine in serum is performed by addition of 0.1 normal iodine followed by stoppage of oxidation by addition of alkaline sulfite to produce certain fluorescence. The induced fluorescence is measured at specific emission wavelength after excitation at another specific wave length that differs according to the type of catecholamine, epinephrine or norepinephrine. The intensity of the fluorescence produced is directly proportional to the concentration of catecholamine in the serum sample [19].
3. Blood carboxyhemoglobin (COHb) was measured by using spectrophotometer (BAUSCH & LOMB Spectronic 2000). Ten *µ*L of blood was added to 20 mL of diluent (2.5 mg/mL sodium dithionite was dissolved in 0.01 mol/L TRIS (hydroxymethyl) amino methane just before use) and its absolute derivative absorption at 420 nm was compared with the absolute derivative value at 420 nm for saturated blood samples (5 mL of diluted blood was saturated by bubbling CO gas for 30 minutes) to give the percentage saturation of COHb [20].

### 2.5. Analysis of Brain Homogenates

The skulls were opened carefully to take part from the brain dissecting it at region of cerebral cortex, used for preparation of tissue homogenates for estimation of tissue MDA and TAC* (Biodiagnostic, Egypt)*. Specimens from brain were weighed and homogenized separately in potassium phosphate buffer 10 mM pH (7.4). The homogenates were centrifuged at 5000 rpm for 10 min at 4°C. The resulting supernatant was used for determination of MDA according to the method of Ohkawa et al. [21] and TAC using colorimetric assay kit according to the manufacturer's instructions* (Biodiagnostic, Egypt)*.

### 2.6. Expression Analyses

Total RNA was purified from homogenized brain specimen using RiboZol RNA extraction reagent (Amresco, Solon, USA) following the manufacturer's instructions. Isolated total RNA was used as template for reverse transcriptase-polymerase chain reaction (RT-PCR) using OneStep RT-PCR Kit (Qiagen, UK). Sequences of the primers used for PCR amplification were as follows: Ngb forward; 5-CTCTGGAAC ATGGCACTGTC-3; reverse, 5-GCACTGGCTCGTCTCTTACT-3; product size 425 bp, for S100B forward; 5-TTGCCCTCATTGATGTCTTCCA-3 and 5-TCTGCCACGGGGAAACGGCTCA-3; product size 296 bp, and for 18s; forward, 5-TTGACGGAAGGGCACCACCAG-3, reverse, 5-GCACCACCACCCACGGAATCG-3; product size 131 bp. RT-PCR products were separated on 2% agarose gel, visualized by ethidium bromide staining. The intensity of the PCR product bands was quantified using gel documentation system software (Biometra GmbH, Germany).

### 2.7. Statistical Analysis

All data were represented as mean ± standard error of the mean (mean ± SEM). Data were analyzed by one-way analysis of variance (ANOVA) followed by the Bonferroni multiple comparison test. A *p* value ≤ 0.05 was considered to indicate statistical significance.

## 3. Results

The results clearly demonstrated that exposure of the rats to WIRS produced a significant increase in the serum catecholamines (epinephrine and norepinephrine) and corticosterone levels in comparison with control group. HO-1 inducer (hemin) produced a significant decrease in epinephrine, norepinephrine, and corticosterone levels when compared to WIRS group but failed to produce significant change when compared to control group (Table 1).

It was found that WIRS significantly increased MDA and decreased TAC in the brain tissue when compared to control rats. Hemin pretreatment produced a significant decrease in MDA and increase in TAC in the brain tissue as compared to WIRS group but failed to alter both significantly when compared to control group (Table 2).

**Figure 1:**
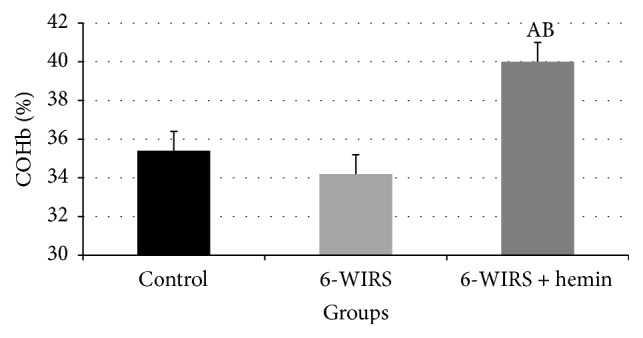
Effect of water immersion restraint stress (WIRS) with or without hemin on carboxyhemoglobin level in male albino rats. ^A^Significant difference from the control group and ^B^significant difference from the WIRS group, *p* ≤ 0.05. Values are expressed as mean ± SEM of 10 rats in each group.

Hemin pretreatment significantly increased blood COHB level in WIRS group to prove that endogenous CO production was modified through modulation of heme oxygenase activity. Exposure of the rats to WIRS failed to alter significantly the blood COHB level (Figure 1).

**Figure 2:**
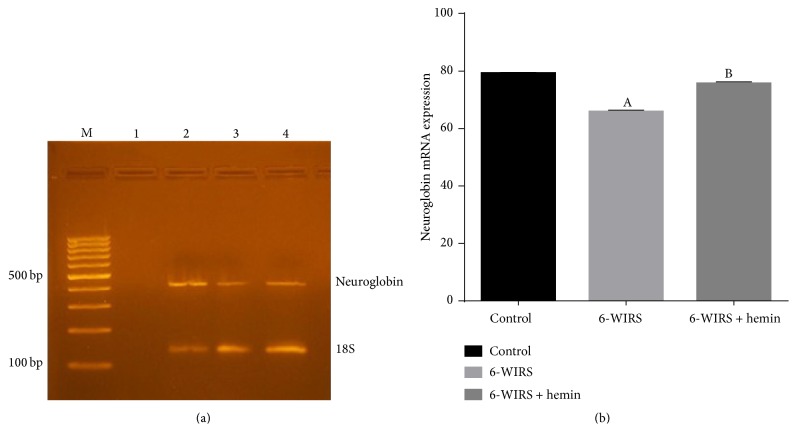
Effect of 6-hour water immersion restraint stress (WIRS) with or without hemin on neuroglobin mRNA expression in brain. (a) M: 100 bp ladder. Lane 1: negative control, lane 2: control group, lane 3: WIRS group, and lane 4: hemin-treated group. (b) ^A^Significant difference from the control group and ^B^significant difference from the WIRS group, *p* ≤ 0.05. Values are expressed as mean ± SEM of 10 rats in each group.

Neuroglobin mRNA expression was significantly reduced in WIRS group compared to control group while hemin injection resulted in significant increase in Ngb expression in brain tissue compared to stress group and it was nonsignificantly different compared to control group (Figure 2).

**Figure 3:**
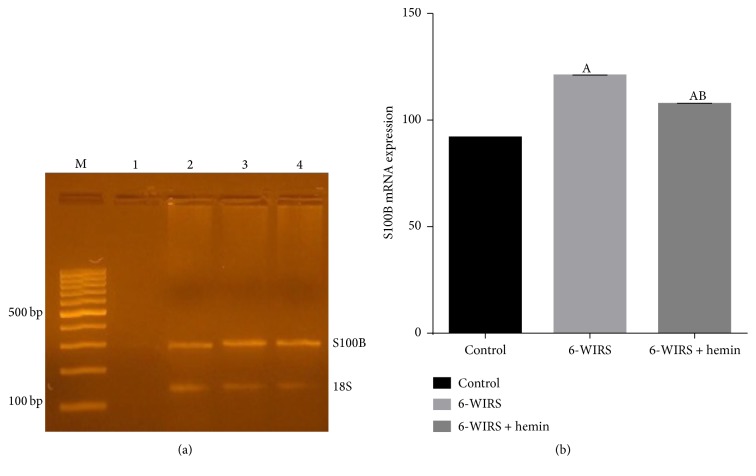
Effect of 6-hour water immersion restraint stress (WIRS) with and without hemin on protein S100B mRNA expression in brain. (a) M: 100 bp ladder. Lane 1: negative control, lane 2: control group, lane 3: WIRS group, and lane 4: hemin-treated group. (b) ^A^Significant difference from the control group and ^B^significant difference from the WIRS group, *p* ≤ 0.05. Values are expressed as mean ± SEM of 10 rats in each group.

The obtained data showed that WIRS significantly increased S100B mRNA expression in comparison with control group. On the other hand, hemin pretreatment significantly decreased S100B mRNA expression in brain tissue as compared to WIRS group but still significantly higher than control group (Figure 3).

## 4. Discussion

In the present study, acute stress markedly increased serum catecholamines (epinephrine and norepinephrine) and corticosterone which is in agree with the findings of Sanchez et al. [22] and Ohta et al. [17]. Other studies have indicated the increase in norepinephrine levels after cold-induced stress [23] and in the activity of tyrosine hydroxylase, a rate-limiting enzyme in catecholamine biosynthesis [24]. Also WIRS could affect the hypothalamo-pituitary-adrenal axis that results in increased plasma glucocorticoids, generally cortisol or corticosterone [25].

Injection of hemin to stressed rats in the present study significantly decreased serum corticosterone, epinephrine, and norepinephrine levels. These results are in agreement with previous studies [26, 27]. Grion et al. [28] found that CO produced from heme degradation binds to the heme group of cytochrome P450 enzyme and inhibits its roles in synthesis of steroids, which in turn inhibits catecholamine synthesis, storage, and secretion [29].

Also Jeong et al. [30] stated that these effects could be mediated by the inhibitory effect of the induced-HO enzyme on nitric oxide synthase activity because nitric oxide has a stimulatory effect on catecholamines biosynthetic enzymes [31].

WIRS causes disruption of nonenzymatic antioxidant defence systems in the brain of rats [32]. Data of the present study clearly demonstrates that acute WIRS significantly increases MDA and decreases TAC which is in agreement with other investigators [33, 34]. Kumar et al. [35] reported that acute restraint stress stimulates numerous cellular cascades that lead to increase in ROS production with oxide vital cellular components, such as lipids, proteins, and DNA, changing their structure and their function which leads to cell damage and even cell death.

The brain is especially vulnerable to oxidative damage because the tissue has high oxygen consumption, high levels of unsaturated fatty acids, and low to moderate levels of antioxidant enzyme activities compared with other tissues [36]. Zaidi and Banu [37] have reported that exposure of rats to 6 h of immobilization causes a decrease in reduced glutathione (GSH) level and an increase in MDA level in the brain. Also previous studies revealed that exposure of rats to 1 h of restraint stress increases MDA levels in the serum and brain and decreases the levels of GSH [38, 39].

Increased corticosterone induces oxidative stress by downregulating the gene expression of glutathione peroxidase, an enzyme that metabolizes hydrogen peroxide in the presence of GSH, and by upregulating the gene expression of NADPH oxidase, an enzyme that generates superoxide radical [40]. It is known that lipid peroxidation in the brain is involved in the pathogenesis of neurodegenerative diseases [41].

In this study, hemin pretreatment produced a significant decrease of MDA and increase of TAC activities in brain as compared with the stressed group which is in agreement with Yang et al. [42]. HO-1 mostly mediates antioxidant effects through different mechanisms as upregulation of the iron storage protein, ferritin [43], production of a lipophilic antioxidant, biliverdin, and induction of nuclear factor erythroid 2-related factor 2 (Nrf2) that activate antioxidant response elements, which regulate genes of many antioxidant enzymes [44].

Ngb was identified and initially described by Burmester et al. [45]. Ngb is an endogenous neuroprotectant and is predominantly expressed in the nervous system [46]. Its functions include binding, storing, and transporting oxygen and scavenging reactive species [47].

In the present work, WIRS induced significant decrease in Ngb expression compared to control group accompanied with increased MDA and decreased TAC. This result is in agreement with Guo et al. [46] who reported reduced endogenous Ngb with increased oxidative stress in cerebral cortex in the neurotoxic conditions.

The effect of acute stressors on Ngb is controversial; increased expression of Ngb has been observed in the brain tissue of patients with ischemic stroke [48]. Also it has been reported that hypoxia through hypoxia-inducible factor-1*α* (HIF-1*α*) positively regulates Ngb expression [49]. Overexpressing Ngb reduced brain infarct size caused by permanent middle artery occlusion [50, 51] and numerous experimental studies demonstrated that overexpression of Ngb effectively counteracted tissue injury induced by experimental ischemia [52, 53]. However, Di Pietro et al. [54] reported that mild traumatic brain injury, although causing a reversible increase in oxidative stress, did not induce any change in neuroglobin but severe traumatic brain injury caused increase in neuroglobin expression.

This converse may be caused by different experimental protocols used in animal strains studied as the expression of Ngb is related to both the severity of the injury and the time of postinjury suggesting that the modulation of this protein might play a significant role in the pathophysiology of brain injury.

The mechanisms underlying improvement of neurological function and prognosis via endogenous Ngb remain unclear; however, many hypotheses exist; Ngb plays an important physiological role in transportation of oxygen to the mitochondria and preserves mitochondria ATP production [8, 55]. Also Ngb protects cells via clearance of reactive oxygen species produced by oxidative stress [54] and it has antiapoptotic effects via reduction of cytochrome c [56].

In the present study, neuroglobin mRNA was significantly reduced in WIRS group compared to control group while hemin injection resulted in significant increase in Ngb from control group.

Like hemoglobin and myoglobin which can be induced by hemin in a dose-dependent manner [57], the structural and functional similarity of Ngb to hemoglobin and myoglobin suggests that Ngb may be a hemin-responsive gene and its expression is mediated by the soluble guanylate cyclase-protein kinase G (sGC-PKG pathway). Protein kinase C (PKC) is also involved in hemin-induced genes expression and erythroid differentiation [58, 59]. In* in vitro* studies of Ngb-neuronal expression, Zhu et al. [58] found that the induction of Ngb mRNA and protein by hemin was concentration and time-dependent.

S100B has been widely used as a marker for neurotrauma, neuroinflammation, and increased BBB permeability [60–62]. Furthermore, S100B is even regarded as a biomarker of brain damage [63]. A previous study revealed that significant level of stress, depression, and anxiety is accompanied by increased blood-brain barrier permeability and increased serum S100B level [64]. Peripheral origins of S100B such as fat, muscle, and marrow have been reported but animal research has suggested the brain to be the main source of the release of S100B [65]. In the current study, S100B mRNA expression in brain tissue is measured and it was shown that there is significant increase in S100B expression in stressed group which may be due to greater stress-induced levels of the proinflammatory mediators IL-6 and TNF*α* [64].

In a previous study it was found that systemic hemin therapy attenuates blood-brain barrier disruption after intracerebral hemorrhage [66] and our study has shown that by giving hemin to stressed animals there was significant decrease in expression of S100B in brain tissue and this may be explained by the fact that hemin is a very potent HO-1 inducer and its benefits may be attributed to its catalytic activity and to the antioxidant and anti-inflammatory properties of the two breakdown products, bilirubin and CO [66].

In conclusion and according to our results, the role of the products of HO enzyme in the stress response could be clarified for its antistress effect. Expression of Ngb is acutely reduced in stressed rats with increased expression of S100B and oxidative stress in the brain. Hemin pretreatment significantly attenuated brain alterations induced by acute stress in rats through increased TAC, increased expression of Ngb, and decreased expression of S100B in brain tissue.

These data contribute to reinforcing the concept that Ngb may represent a novel target for the pharmacological therapy of various neurological disorders and it is important to determine which mechanisms are important for Ngb-mediated neuroprotection. Using gene therapy or natural inducers of HO as reported by Li Volti et al. [67] is preferred in future studies. A possible limitation of the current study is assessment of the markers on specific subregions of the brain and measurement of the proinflammatory mediators, IL-6 and TNF*α*, which would further increase the validity of the results.

## Figures and Tables

**Table 1:** Effect of 6-hour water immersion restraint stress (WIRS) with or without hemin on serum catecholamines (epinephrine and norepinephrine) and corticosterone in male albino rats.

Parameters	Groups		
	Control	WIRS	WIRS + hemin
Epinephrine (ng/mL)	84.5 ± 3.9	108 ± 2.4^A^	88 ± 4.2^B^
Norepinephrine (ng/mL)	184.8 ± 5.6	220 ± 7.2^A^	188.3 ± 5.8^B^
Corticosterone (*μ*g/mL)	37.3 ± 1.7	44.9 ± 1.8^A^	38 ± 0.8^B^

^A^Significant difference from the control group and ^B^significant difference from the WIRS group, *p* ≤ 0.05. Values are expressed as mean ± SEM of 10 rats in each group.

**Table 2:** Effect of 6-hour water immersion restraint stress (WIRS) with or without hemin on brain oxidative state in male albino rats.

Parameters	Groups		
	Control	WIRS	WIRS + hemin
Brain MDA (pg/mg tissue)	3.5 ± 0.2	6.2 ± 0.07^A^	3.8 ± 0.1^B^
Brain TAC (*μ*M/mg tissue)	7.2 ± 0.3	4.2 ± 0.2^A^	6.9 ± 0.2^B^

MDA: malondialdehyde and TAC: total antioxidant capacity. ^A^Significant difference from the control group and ^B^significant difference from the WIRS group, *p* ≤ 0.05. Values are expressed as mean ± SEM of 10 rats in each group.

## References

[1] Amin S. N., El-Aidi A. A., Ali M. M., Attia Y. M., Rashed L. A. (2015). Modification of hippocampal markers of synaptic plasticity by memantine in animal models of acute and repeated restraint stress: implications for memory and behavior. *NeuroMolecular Medicine*.

[2] Khalaj L., Nejad S. C., Mohammadi M. (2013). Gemfibrozil pretreatment proved protection against acute restraint stress-induced changes in the male rats' hippocampus. *Brain Research*.

[3] Farooqui T., Farooqui A. A. (2011). Lipid-mediated oxidative stress and inflammation in the pathogenesis of Parkinson's disease. *Parkinson's Disease*.

[4] Chen J. (2014). Heme oxygenase in neuroprotection: from mechanisms to therapeutic implications. *Reviews in the Neurosciences*.

[5] Justicia C., Ramos-Cabrer P., Hoehn M. (2008). MRI detection of secondary damage after stroke: chronic iron accumulation in the thalamus of the rat brain. *Stroke*.

[6] Jeong G. S., Lee D. S., Kim D. C. (2011). Neuroprotective and anti-inflammatory effects of mollugin via up-regulation of heme oxygenase-1 in mouse hippocampal and microglial cells. *European Journal of Pharmacology*.

[7] Tronel C., Rochefort G. Y., Arlicot N., Bodard S., Chalon S., Antier D. (2013). Oxidative stress is related to the deleterious effects of heme oxygenase-1 in an *in vivo* neuroinflammatory rat model. *Oxidative Medicine and Cellular Longevity*.

[8] Yu Z., Poppe J. L., Wang X. (2013). Mitochondrial mechanisms of neuroglobin's neuroprotection. *Oxidative Medicine and Cellular Longevity*.

[9] Götting M., Nikinmaa M. (2015). More than hemoglobin—the unexpected diversity of globins in vertebrate red blood cells. *Physiological Reports*.

[10] Song X., Xu R., Xie F., Zhu H., Zhu J., Wang X. (2014). Hemin offers neuroprotection through inducing exogenous neuroglobin in focal cerebral hypoxic-ischemia in rats. *International Journal of Clinical and Experimental Pathology*.

[11] Zhang B., Ji X., Zhang S. (2013). Hemin-mediated neuroglobin induction exerts neuroprotection following ischemic brain injury through PI3K/Akt signaling. *Molecular Medicine Reports*.

[12] Jauregui-Huerta F., Ruvalcaba-Delgadillo Y., Gonzalez-Perez O., Gonzalez-Castañeda R., Garcia-Estrada J., Luquin S. (2010). Responses of glial cells to stress and glucocorticoids. *Current Immunology Reviews*.

[13] Undén J., Ingebrigtsen T., Romner B. (2013). Scandinavian guidelines for initial management of minimal, mild and moderate head injuries in adults: an evidence and consensus-based update. *BMC Medicine*.

[14] Zongo D., Ribéreau-Gayon R., Masson F. (2012). S100-B protein as a screening tool for the early assessment of minor head injury. *Annals of Emergency Medicine*.

[15] Ndisang J. F., Wu L., Zhao W., Wang R. (2003). Induction of heme oxygenase-1 and stimulation of cGMP production by hemin in aortic tissues from hypertensive rats. *Blood*.

[16] Xie F., Xu R., Song X., Zhu H., Wang X., Zhu J. (2014). Joint protective effect of exogenous neuroglobin and hemin in rat focal ischemic brain tissues. *International Journal of Clinical and Experimental Medicine*.

[17] Ohta Y., Yashiro K., Kaida S., Imai Y., Ohashi K., Kitagawa A. (2013). Water-immersion restraint stress disrupts nonenzymatic antioxidant defense systems through rapid and continuous ascorbic acid depletion in the adrenal gland of rats. *Cell Biochemistry and Function*.

[18] Mattingly D. (1962). Practical procedure for estimation of corticosterone or hydrocortisone. *Journal of Clinical Pathology*.

[19] Ciarlone A. E. (1978). Further modification of a fluorometric method for analyzing brain amines. *Microchemical Journal*.

[20] Mayes R. W. (1993). Measurement of carbon monoxide and cyanide in blood. *Journal of Clinical Pathology*.

[21] Ohkawa H., Ohishi N., Yagi K. (1979). Assay for lipid peroxides in animal tissues by thiobarbituric acid reaction. *Analytical Biochemistry*.

[22] Sanchez A., Toledo-Pinto E. A., Menezes M. L., Pereira O. C. M. (2003). Changes in norepinephrine and epinephrine concentrations in adrenal gland of the rats submitted to acute immobilization stress. *Pharmacological Research*.

[23] Dronjak S., Jezova D., Kvetnansky R. (2004). Different effects of novel stressors on sympathoadrenal system activation in rats exposed to long-term immobilization. *Annals of the New York Academy of Sciences*.

[24] Xu L., Chen X., Sun B., Sterling C., Tank A. W. (2007). Evidence for regulation of tyrosine hydroxylase mRNA translation by stress in rat adrenal medulla. *Brain Research*.

[25] Montoro J., Mullol J., Jáuregui I. (2009). Stress and allergy. *Journal of Investigational Allergology and Clinical Immunology*.

[26] Asif A. R., Ljubojevic M., Sabolic I. (2006). Regulation of steroid hormones biosynthesis and organic anion transporters by forskolin and DHEA-S treatment in adrenocortical cells. *American Journal of Physiology. Endocrinology and Metabolism*.

[27] El-Sayed S., Hassan M., Ibrahim M., Elbassuoni E., Aziz N. (2012). Modified endogenous carbon monoxide production through modulation of heme oxygenase activity alters some aspects of the cold restraint stress response in male albino rats. *Endocrine Regulations*.

[28] Grion N., Repetto E. M., Pomeraniec Y. (2007). Induction of nitric oxide synthase and heme oxygenase activities by endotoxin in the rat adrenal cortex: involvement of both signaling systems in the modulation of ACTH-dependent steroid production. *Journal of Endocrinology*.

[29] Hodel A. (2001). Effects of glucocorticoids on adrenal chromaffin cells. *Journal of Neuroendocrinology*.

[30] Jeong S., Son Y., Lee J. (2015). Resveratrol analog piceatannol restores the palmitic acid-induced impairment of insulin signaling and production of endothelial nitric oxide via activation of anti-inflammatory and antioxidative heme oxygenase-1 in human endothelial cells. *Molecular Medicine Reports*.

[31] Kim D., Choi H. J., Kim S. W., Cho S.-W., Hwang O. (2003). Upregulation of catecholamine biosynthetic enzymes by nitric oxide. *Journal of Neuroscience Research*.

[32] Ohta Y., Yashiro K., Ohashi K., Imai Y. (2012). Disruption of non-enzymatic antioxidant defense systems in the brain of rats with water-immersion restraint stress. *Journal of Clinical Biochemistry and Nutrition*.

[33] Dal Santo G., Conterato G. M. M., Barcellos L. J. G., Rosemberg D. B., Piato A. L. (2014). Acute restraint stress induces an imbalance in the oxidative status of the zebrafish brain. *Neuroscience Letters*.

[34] Cai J., Cao S., Chen J., Yan F., Chen G., Dai Y. (2015). Progesterone alleviates acute brain injury via reducing apoptosis and oxidative stress in a rat experimental subarachnoid hemorrhage model. *Neuroscience Letters*.

[35] Kumar A., Garg R., Prakash A. K. (2010). Effect of St. John's Wort (*Hypericum perforatum*) treatment on restraint stress-induced behavioral and biochemical alteration in mice. *BMC Complementary and Alternative Medicine*.

[36] Halliwell B. (2006). Oxidative stress and neurodegeneration: where are we now?. *Journal of Neurochemistry*.

[37] Zaidi S. M. K. R., Banu N. (2004). Antioxidant potential of vitamins A, E and C in modulating oxidative stress in rat brain. *Clinica Chimica Acta*.

[38] Pal R., Gulati K., Chakraborti A., Banerjee B., Ray A. (2006). Role of free radicals in stress-induced neurobehavioural changes in rats. *Indian Journal of Experimental Biology*.

[39] Gulati K., Chakraborti A., Ray A. (2009). Differential role of nitric oxide (NO) in acute and chronic stress induced neurobehavioral modulation and oxidative injury in rats. *Pharmacology Biochemistry and Behavior*.

[40] You J.-M., Yun S.-J., Nam K. N., Kang C., Won R., Lee E. H. (2009). Mechanism of glucocorticoid-induced oxidative stress in rat hippocampal slice cultures. *Canadian Journal of Physiology and Pharmacology*.

[41] Adibhatla R. M., Hatcher J. F. (2010). Lipid oxidation and peroxidation in CNS health and disease: from molecular mechanisms to therapeutic opportunities. *Antioxidants and Redox Signaling*.

[42] Yang Y., Wang J., Li Y. (2015). HO-1 signaling activation by pterostilbene treatment attenuates mitochondrial oxidative damage induced by cerebral ischemia reperfusion injury. *Molecular Neurobiology*.

[43] Reed J. R., Gallelli L. (2012). Elucidating the role of biliverdin reductase in the expression of heme oxygenase-1 as a cytoprotective response to stress. *Pharmacology*.

[44] Kang J. S., Choi I. W., Han M. H. (2015). The cytoprotective effect of petalonia binghamiae methanol extract against oxidative stress in C2C12 myoblasts: mediation by upregulation of heme oxygenase-1 and nuclear factor-erythroid 2 related factor 2. *Marine Drugs*.

[45] Burmester T., Welch B., Reinhardt S., Hankeln T. (2000). A verteblrate globin expressed in the brain. *Nature*.

[46] Guo Y., Yuan H., Jiang L. (2015). Involvement of decreased neuroglobin protein level in cognitive dysfunction induced by 1-bromopropane in rats. *Brain Research*.

[47] He A. W., Yang T., Chen S. Q. (2011). Effects of Hemin on neuroglobin expression after cardiopulmonary resuscitation in rats. *World Journal of Emergency Medicine*.

[48] Jin K., Mao Y., Mao X., Xie L., Greenberg D. A. (2010). Neuroglobin expression in ischemic stroke. *Stroke*.

[49] Haines B., Demaria M., Mao X. (2012). Hypoxia-inducible factor-1 and neuroglobin expression. *Neuroscience Letters*.

[50] Raida Z., Hundahl C. A., Nyengaard J. R., Hay-Schmidt A. (2013). Neuroglobin over expressing mice: expression pattern and effect on brain ischemic infarct size. *PLoS ONE*.

[51] Shang A., Yang Y., Wang H. (2015). Upregulation of neuroglobin expression and changes in serum redox indices in a rat model of middle cerebral artery occlusion. *Molecular Medicine Reports*.

[52] Yu Z., Liu J., Guo S. (2009). Neuroglobin-overexpression alters hypoxic response gene expression in primary neuron culture following oxygen glucose deprivation. *Neuroscience*.

[53] Guo Z. D., Sun X. C., Zhang J. H. (2011). Mechanisms of early brain injury after SAH: matrix metalloproteinase 9. *Acta Neurochirurgica Supplement*.

[54] Di Pietro V., Lazzarino G., Amorini A. M. (2014). Neuroglobin expression and oxidant/antioxidant balance after graded traumatic brain injury in the rat. *Free Radical Biology and Medicine*.

[55] Yu Z., Zhang Y., Liu N. (2015). Roles of neuroglobin binding to mitochondrial complex III subunit cytochrome *c*1 in oxygen-glucose deprivation-induced neurotoxicity in primary neurons. *Molecular Neurobiology*.

[56] Lin Y., Cai B., Xue X., Fang L., Wu Z., Wang N. (2015). TAT-mediated delivery of neuroglobin attenuates apoptosis induced by oxygen-glucose deprivation via the Jak2/Stat3 pathway *in vitro*. *Neurological Research*.

[57] Dean A., Ley T. J., Humphries R. K., Fordis M., Schechter A. N. (1983). Inducible transcription of five globin genes in K562 human leukemia cells. *Proceedings of the National Academy of Sciences of the United States of America*.

[58] Zhu Y., Sun Y., Jin K., Greenberg D. A. (2002). Hemin induces neuroglobin expression in neural cells. *Blood*.

[59] Jin K., Mao X. O., Xie L., John V., Greenberg D. A. (2011). Pharmacological induction of neuroglobin expression. *Pharmacology*.

[60] Marchi N., Cavaglia M., Fazio V., Bhudia S., Hallene K., Janigro D. (2004). Peripheral markers of blood-brain barrier damage. *Clinica Chimica Acta*.

[61] van Munster B. C., Korse C. M., de Rooij S. E., Bonfrer J. M., Zwinderman A. H., Korevaar J. C. (2009). Markers of cerebral damage during delirium in elderly patients with hip fracture. *BMC Neurology*.

[62] Vos P. E., Lamers K. J. B., Hendriks J. C. M. (2004). Glial and neuronal proteins in serum predict outcome after severe traumatic brain injury. *Neurology*.

[63] Schiavi P., Iaccarino C., Servadei F. (2012). The value of the calcium binding protein S100 in the management of patients with traumatic brain injury. *Acta Biomedica*.

[64] Li X., Wilder-Smith C. H., Kan M. E., Lu J., Cao Y., Wong R. K. (2014). Combat-training stress in soldiers increases S100B, a marker of increased blood-brain-barrier permeability and induces immune activation. *Neuroendocrinology Letters*.

[65] Lipcsey M., Olovsson M., Larsson E. (2010). The brain is a source of S100B increase during endotoxemia in the pig. *Anesthesia and Analgesia*.

[66] Lu X., Chen-Roetling J., Regan R. F. (2014). Systemic hemin therapy attenuates blood-brain barrier disruption after intracerebral hemorrhage. *Neurobiology of Disease*.

[67] Li Volti G., Sacerdoti D., Di Giacomo C. (2008). Natural heme oxygenase-1 inducers in hepatobiliary function. *World Journal of Gastroenterology*.

